# Thermal tolerance and fish heart integrity: fatty acids profiles as predictors of species resilience

**DOI:** 10.1093/conphys/coaa108

**Published:** 2020-12-26

**Authors:** Felix Christen, France Dufresne, Gabriel Leduc, Bernard A Dupont-Cyr, Grant W Vandenberg, Nathalie R Le François, Jean-Claude Tardif, Simon G Lamarre, Pierre U Blier

**Affiliations:** 1 Département de Biologie, Université du Québec à Rimouski, Rimouski, Québec, G5L3A1, Canada; 2 Département de Sciences Animales, Université Laval, Québec, Québec, G1V 0A6, Canada; 3 Biodôme de Montréal, Montréal, Québec, H1V 1B3, Canada; 4 Montreal Heart Institute, Université de Montréal, Montréal, Québec, H1T 1C8, Canada; 5 Département de Biologie, Université de Moncton, Moncton, New-Brunswick, E1A 3E9, Canada

**Keywords:** CTmax, fatty acids, fish heart, oxidative stress, temperature

## Abstract

The cardiovascular system is a major limiting system in thermal adaptation, but the exact physiological mechanisms underlying responses to thermal stress are still not completely understood. Recent studies have uncovered the possible role of reactive oxygen species production rates of heart mitochondria in determining species’ upper thermal limits. The present study examines the relationship between individual response to a thermal challenge test (CT_max_), susceptibility to peroxidation of membrane lipids, heart fatty acid profiles and cardiac antioxidant enzyme activities in two salmonid species from different thermal habitats (*Salvelinus alpinus*, *Salvelinus fontinalis*) and their hybrids. The susceptibility to peroxidation of membranes in the heart was negatively correlated with individual thermal tolerance. The same relationship was found for arachidonic and eicosapentaenoic acid. Total H_2_O_2_ buffering activity of the heart muscle was higher for the group with high thermal resistance. These findings underline a potential general causative relationship between sensitivity to oxidative stress, specific fatty acids, antioxidant activity in the cardiac muscle and thermal tolerance in fish and likely other ectotherms. Heart fatty acid profile could be indicative of species resilience to global change, and more importantly the plasticity of this trait could predict the adaptability of fish species or populations to changes in environmental temperature.

## Teaser

The capacity of ectotherms to adapt to new thermal habitat depends on the physiological impact of changes in temperature. We observed here that heart tolerance to oxidative stress, dictated by membrane fatty acid profile, might be a significant driver in setting upper thermal limits in Arctic charr.

## Introduction

Global temperatures are rising (Brown & Caldeira, 2017), resulting in the occurrence of periodic extreme heat waves (Gerald and Tebaldi, 2004; Diffenbaugh and Field, 2014) and changes in species distribution patterns (Iftikar et al., 2014; Sunday et al., 2012). Temperature changes have intense effects on biochemical reaction rates of all organisms, notably ectothermic species such as fish (Hochachka and Somero, 1968; Fry, 1971). Previous studies have suggested that the heart could be the critical limiting systems in temperature response (reviewed in Eliason and Anttila, 2017) due to the high thermal sensitivity of mitochondrial metabolism and to the fact that vertebrate heart functions rely on tissue aerobic capacity. The heat-induced heart failure has been shown to be correlated to the critical upper-temperature limit (CT_max_) (Farrell et al., 2009, 1996; Somero, 2002) but the exact physiological mechanisms limiting fish capacity to face acute temperature increases are still not completely understood (Clark et al., 2013).

Some authors have suggested that oxygen transport could limit the thermal tolerance of ectotherms, as a mismatch occurs between the capacity to supply oxygen and the demand of highly active tissues when approaching extreme temperatures (Pörtner et al., 2017; Pörtner & Knust, 2007). Oxygen might, however, be more available at high temperatures since oxygen diffusion rates increase with increasing temperatures (Verberk et al., 2011). In this case, the mismatch would result from a higher increase in demand than in supply capacity. Experimental evidence suggests that oxygen availability does not directly set upper thermal limits in different fish species (Devor *et al.*, 2015; Ern *et al.*, 2016, 2017). Heart mitochondria have been put forward as potential causative agents of heat-induced heart failure as decline in their efficiency, and the plateauing of respiration rate are observed as temperature gets closer to thermal limits (Iftikar and Hickey, 2013; Blier *et al.*, 2014; Iftikar *et al.*, 2014; Ekström *et al.*, 2017).

Iftikar and Hickey (2013) found a significant decrease in ATP production in heart myofibers from the New Zealand fish, *Notolabrus celidotus*, and more importantly of the efficiency (ATP/O_2_) at the critical temperature of the fish. In Arctic charr (AC), *Salvelinus alpinus*, Christen et al. (2018) showed that the respiration rate of heart mitochondria saturated with substrates stops increasing when temperature gets close to CT_max,_ while reactive oxygen species (ROS) efflux significantly increases. These two independent studies underline the potential role of ROS in heat-induced heart failure. In different animal models, mitochondrial functions, catalytic capacities and regulation have been associated to membrane structure and composition and, in particular, to fatty acid profile as well as to the susceptibility of tissue or mitochondrial membrane to ROS assaults (Hulbert *et al.*, 2007; Munro and Blier, 2012; Munro *et al.*, 2013; Blier *et al.*, 2017).

Cellular and mitochondrial membrane composition of fish, especially salmonids, are rich in polyunsaturated fatty acids (PUFAs) (Almaida-Pagán *et al.*, 2014; Sissener *et al.*, 2020). Fatty acid profiles of cardiac tissue are associated with traits such as critical swimming speed and growth in seabass (*Dicentrarchus labrax)* and Atlantic salmon (*Salmo salar*), respectively (McKenzie *et al.*, 1998; Chatelier *et al.*, 2006). Long-term acclimation of species to different thermal habitats affected the fatty acid composition of the heart, i.e. a significant decrease of omega 3(ω-3) and a concomitant increase of saturated fatty acids occurred at the highest acclimation temperature were observed (Ekström *et al.*, 2017). High PUFA content in heart mitochondria membranes is susceptible to increase oxidative stress (Lemieux *et al.*, 2011) and lipid peroxidation (Else, 2017; Hulbert *et al.*, 2017). Moreover, this susceptibility of lipids to peroxidation is argued to be mechanistically linked to lifespan (Munro and Blier, 2012). Longevity and peroxidation index (PI) of membranes are also negatively correlated in mammals and birds (Hulbert *et al.*, 2007). The association between membrane robustness, modulated by fatty acid profile, and physiological performance or longevity makes us suspect that the upper thermal limit (CTmax) is potentially related to membrane robustness against oxidation. This mechanistic link is even more likely since at a temperature close to the upper limits, excessive ROS efflux was detected in fish heart mitochondria (Christen *et al.*, 2018).

Our study examined the potential link between CT_max_ and oxidative stress tolerance expressed by fatty acid profile in the heart. We hypothesized that susceptibility to oxidative stress, and therefore to temperature increases, would be higher in individuals with high proportions of highly peroxidable fatty acids. In addition, we evaluated the antioxidant activity of cardiac muscle fibers to test whether heat-tolerant individuals display a better antioxidant defense than less tolerant individuals. Linking structural or metabolic traits to tolerance to temperature increases advocates for further experimentation to test if the variability of these traits can be inherited along with CT_max_, allowing adaptation to environmental temperatures increases in fish.

In order to maximize detectable divergence in thermal performance, antioxidant activity and fatty acid profiles, we chose two species with contrasting geographical distribution patterns: AC with a holarctic distribution and a narrower temperature range (4–16°C, Fishbase.org) and brook charr (BC; *Salvelinus fontinalis*) with a more southern distribution and a more broad range of temperatures (0–25°C, Fishbase.org). Reciprocal F1 hybrids were included in the experimental design since mitochondrial DNA is associated with metabolic performance and thermal sensitivity (Pichaud *et al.*, 2012, 2013; Baris *et al.*, 2016, 2017). As the mitochondrial genome operates in a specific nuclear background, any disruption of such a mitonuclear coadaptation can lead to a hybrid breakdown (Burton *et al.*, 2013). The potential dysfunction of mitochondria associated to the disruption of mitonuclear coadaptation could have led to elevated levels of oxidative stress in some hybrid individuals (Barreto and Burton, 2013; Pichaud *et al.*, 2019). In a previous study, we have shown that ROS production increases extensively close to thermal limits (Christen *et al.*, 2018). Using reciprocal hybrids to induce a dysfunction of mitochondrial metabolism and exacerbate oxidative stress and potential damage to membranes during the thermal challenge should increase the chances to detect a link between CT_max_ and robustness of membranes to oxidation or peroxidation. Furthermore, if the heart fatty acid profile is associated with CT_max_, we predict that divergences in CT_max_ among the four groups will parallel the divergences in these profiles.

## Materials and methods

### Fish

AC (*S. alpinus*), BC (*S. fontinalis*) and their reciprocal hybrids AC ♀ x BC ♂ (hybrid arctic, HA) and AC ♂ x BC ♀ (hybrid brook, HB) were provided by Pisciculture-des- Monts-de-Bellechase Inc. (St-Damien-de-Buckland, QC, Canada) and Aquaculture Gaspésie Inc. (Gaspé, QC, Canada). The mean fish weight were 421.51 ± 55.25 g (AC), 489.73 ± 38.52 g (BC), 301.76 ± 50.8 g (HA) and 271.87 ± 40.11 g (HB). Fish were transported to the Université du Québec à Rimouski’s aquatic facilities and placed in flow-through 190 L rectangular tanks under natural photoperiod and fed *ad libitum*. Dechlorinated freshwater was supplied at 10°C with a renewal rate of 20%/h prior to the initiation of the experiments. Fish were kept at the facility for at least four weeks and marked individually by using intramuscular passive integrated transponders (PIT-Tag, Biomark, ID, USA). Fish were fed daily *ad libitum* but not fed 24 h before sampling. All experiments were authorized by the local animal ethics committee of the Université du Québec à Rimouski under the Canadian Council on Animal Care.

### Temperature challenge test

The protocol for temperature challenge test (TCT) was adapted from Roze et al. (2013). The tests were conducted in 400 L rearing tanks with all species combined. The challenge started with a rapid temperature increase over 2.5 hours (10 to 20°C) followed by a steadier increase (approximately 2°C per hour) until the end of the experiment. Fish (N, AC = 18, BC = 17, HA = 16 and HB = 20) were immediately removed after loss of equilibrium was observed (fish floating upside down) and rapidly transferred to the initial tank at acclimation temperature, for recovery for at least two weeks before the subsequent analysis (zero mortality recorded). Fish were identified (PIT-tag reading) and the corresponding time and temperature (CT_max_) recorded. The water temperature of the experimental tank was controlled with two TECO TR15 Aquarium chillers (TECO, Ravenna, Italy) equipped with 400 W heater kits. Two EHEIM universal 300 pumps (EHEIM, Deizisau, Germany) supplied water to the heaters and ensured water homogeneity. Oxygen concentration was sustained by extensive air bubbling and kept above 80% air saturation throughout the whole experiment. Two weeks following challenge test, fish were sacrificed by cerebral dislocation and tissues were extracted for analysis.

### Fatty acid profiles of ventricular muscle

Our fatty acid protocol was adapted from Lepage and Roy (1984) and Ekström et al. (2017). At least 50 mg of heart tissue (N ~ 15 per strain) was homogenized in 100 mM potassium phosphate buffer (1 mM EDTA, pH 7.5) and spiked with 0.1 mg of internally added tridecanoic and tricosanoic acid (Nu-Check Prep, Elysian MN, USA). Direct acid-catalyzed trans-methylation was performed by adding 3 ml of 3% sulfuric acid methanol solution at 90°C for 1 h. In order to prepare fatty acid methyl esters (FAME), samples were cooled to 4°C, 5 ml of H_2_O and 1 ml of hexane were added. The sample was vortexed and centrifuged at 3000 *g* for 10 min at room temperature. Hexane was evaporated, and the sample was suspended in 100 μl toluene before injection. FAMEs were separated and quantified by gas chromatography (Trace Ultra 100, Thermo Fisher Scientific, Waltham, MA, USA) equipped with a 60 m x 0.25 mm i.d. capillary column (DB-23, Agilent Technologies Canada, Mississauga, ON, Canada). Helium was used as carrier gas (230 kPa constant pressure), and temperature vaporization was set at 230°C with a split injection of 100 ml min^−1^. Temperature programming was from 50 to 140°C (25°C min^−1^), 140 to 195°C (3°C min^−1^) and a final increase of 4°C min^−1^ up until 225°C maintained for 5 min. Individual methyl esters were identified by comparison with known standards. All chemicals were purchased from Sigma-Aldrich unless otherwise mentioned.

### Enzyme activity

Tissues were homogenized using the following buffer: Trizma 100 mM, Triton X100 0.1%, pH 7.8 prior to enzymatic activity assays. The homogenates were centrifuged at 1500 *g* for 5 min at 4°C, and the supernatant was collected for analysis. Enzyme assays were conducted using a microplate reader PowerWave XS2 (Biotek, Highland Park, USA) in a cold room kept at 15°C. Specific assay conditions were the following:

### Superoxide dismutase

SOD’s assay was adapted from Malstrom et al. (1975). Superoxide dismutase (SOD) was compared to a standard curve of known SOD (Sigma S5395) concentrations in the following reaction medium: Trisma 45 mM, diethylenetriaminepentaacetic acid 0.08 mM, Hypoxanthine 0.08 mM, phosphate potassium 4 mM, Xanthine oxidase 0.0326 U/ml (substrate), Radical detector (Cayman chemical) 0.002%, Tween 20 0.004%. After a 30-minute incubation period, the reading was performed at 450 nm.

### Catalase

Catalase (CAT) activity was estimated as total hydrogen peroxide scavenging capacity of the sample by following H_2_O_2_ removal at 240 nm for 1 minute, assuming a 43.6 M^−1^cm^−1^extinction coefficient using a spectrophotometer (Ultrospec 2100 pro). The reaction medium was: Phosphate potassium 100 mM, H_2_O_2_ 60 mM, Triton X100 0.1%, pH 7.5 (Orr and Sohal, 1992).

### Glutathione peroxidase

Glutathione peroxidase (GPx) was measured by observing nicotinamide adenine dinucleotide phosphate (NADPH) removal at 340 nm, assuming a 6.2 mM^−1^ cm^−1^ extinction coefficient, in the following reaction medium: phosphate potassium, 100 mM; ethylenediaminetetraacetic acid (EDTA), 0.1 mM; sodium azide, 3 mM; NADPH, 0.2 mM; gluthation (GSH), 0.2 mM; tert-butylhydroperoxide, 0.5 mM; glutathione reductase, 0.24 U/ml; Triton X100, 0.2%; pH 7.6. A control without the tert-butylhydroperoxide was used (Munro *et al.*, 2016).

### Glutathione disulfide reductase

Glutathione disulfide reductase (GSR) was measured by observing NADPH removal at 340 nm, assuming a 6.2 mM^−1^cm^−1^ extinction coefficient in the following reaction medium: phosphate potassium, 100 mM; EDTA, 0.1 mM; NADPH, 0.1 mM; GSSG, 2 mM; Triton X100, 0.2%; pH 7.6. A control without GSSG was used (Munro et al., 2016).

### Protein concentration

Protein concentration was determined by using the bicinchoninic acid protein assay (Smith *et al.*, 1985).

### Statistical analysis

PI and unsaturation index (UI) were calculated according to Hulbert et al. (2007). The values are commonly presented without units:

$$\begin{align*}\textrm{PI} = &(0.025 \times \% \ \textrm{monoenoics}) + (1 \times \% \ \textrm{dienoics}) \\&+ (2 \times \% \ \textrm{trienoics}) + (4 \times \% \ \textrm{tetraenoics})\\&+ (6 \times \% \ \textrm{pentaenoics}) + (8 \times \% \ \textrm{hexaenoics})\end{align*}$$

$$\begin{align*}\textrm{UI} = &(1 \times \% \ \textrm{monoenoics}) + (2 \times \% \ \textrm{dienoics}) \\&+ (3 \times \% \ \textrm{trienoics}) + (4 \times \% \ \textrm{tetraenoics})\\&+ (5 \times \% \ \textrm{pentaenoics}) + (6 \times \% \ \textrm{hexaenoics})\end{align*}$$

In order to generate mass-independent data for the time it takes for the fish to loose equilibrium following temperature increases (tCT_max_), PI, eicosapentaenoic acid (EPA), arachidonic acid (ARA) and total n-3-content (N3) residuals were calculated from least square linear regression analysis on body mass. Relationships between tCT_max_ and the various variables were established using linear regression analysis and also analyzed using general linear models (GLMs) with mass as a covariate and AC, BC, HA and HB as group identity in order to estimate to which extent thermal tolerance was associated to fatty acids, PI or antioxidant enzymes activity, independently of group or mass. No interaction was detected between mass, group and time. Differences in mean values of fatty acid profiles, morphological parameters and antioxidant activity were calculated using a one-way analysis of variance (*P* < 0.05) followed by a Tukey HSD test. tCT_max_ and CT_max_ differences among groups were tested using Welch’s ANOVA for unequal variances. All values are reported as mean ± SEM. All statistical tests have been performed using R (R Core Team (2013). R: A language and environment for statistical computing. R Foundation for Statistical Computing, Vienna, Austria. URL http://www.R-project.org/).

## Results

### Temperature challenge test and mass effect

**Fig 1:**
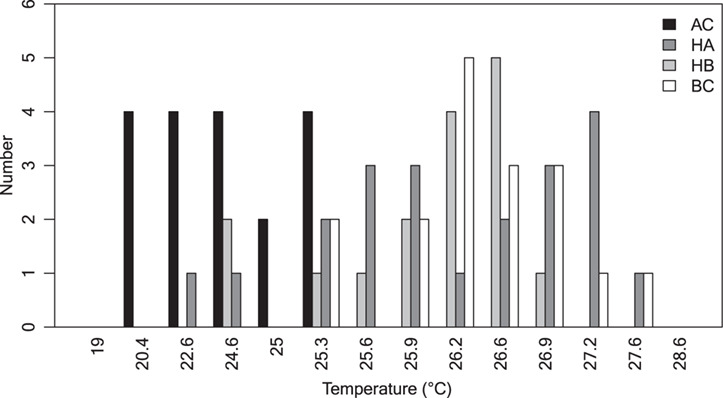
Frequency distribution of CT_max_ (temperature at which fish lose equilibrium) of the four experimental groups. Arctic charr (AC, black bars), hybrid arctic (HA, dark grey bars), HB (light grey bars) and Brook charr (BC, white bars). *N* = 18, 21, 16 and 17, respectively.

**Fig 2:**
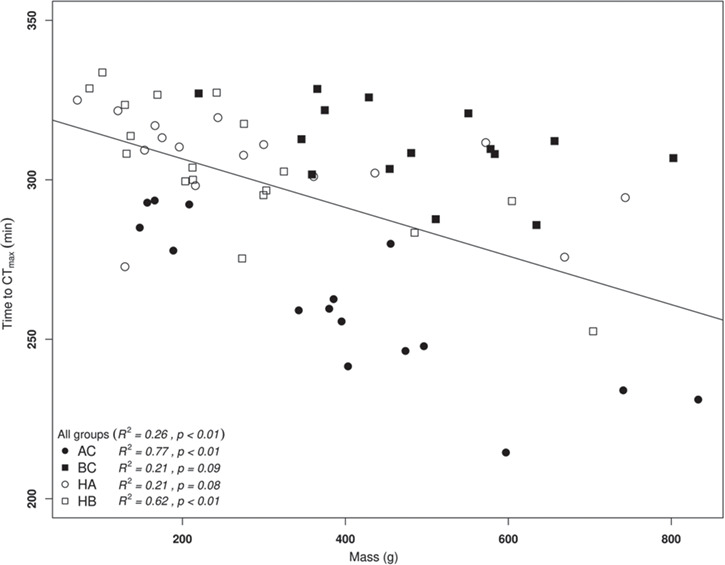
Relationship between body mass (g) and tCT_max_ (min). AC (full circles), BC (full squares), HA (empty circles) and HB (empty squares). The full line represents linear regression for all groups. Equation for all groups: tCT_max_ (min) = −0.08x + 321.8; for AC: tCT_max_ (min) = −0.12x + 306.28; BC: tCT_max_ (min) = −0.04x + 330.27; HA: tCT_max_ (min) = −0.03x + 315.65; HB: tCT_max_ (min) = −0.1x + 330.78.

BC had a significantly higher mean body mass than both hybrids (Table 1). Heart mass did not differ among the two species and their hybrids (Table 1). AC was the least heat-tolerant group with the lowest mean CT_max_, values ranging between 20°C and 25.3°C compared to the three other groups which had higher mean CT_max_ and values ranging between 22°C and 27.6°C (Fig 1, Table 1). CT_max_ values did not differ among BC and the hybrids. Body mass was negatively correlated with time to CT_max_ in AC and HB (Fig 2, linear regression; *P* < 0.01 for both strains) but not in BC and HA. This negative relationship between CT_max_ and body mass prevailed when all four groups were included (Fig 2, *P* < 0.01).

**Table 1:** Mean values of phenotypic traits and CT_max_ among the four groups

Group															
Parameter	AC				BC				HA				HB			
CT_max_ (°C)	23.58	±	0.34	a	26.19	±	0.16	b	25.91	±	0.19	b	25.88	±	0.23	b
tCT_max_ (min)	256.48	±	7.18	a	310.70	±	3.35	b	305.26	±	3.94	b	304.54	±	4.92	b
Mass (g)	421.51	±	53.25	a,b	489.73	±	38.53	b	283.73	±	50.78	a	271.87	±	40.11	a
Heart mass (g)	0.59	±	0.07	a	0.54	±	0.05	a	0.38	±	0.07	a	0.38	±	0.05	a
N	17				15				16				18			

Values are mean ± SEM. CTmax, critical maximum temperature; tCTmax, time to CTmax; dissimilar letters indicate significant differences (*P* < 0,05) between groups.

### Fatty acid profiles, antioxidant enzymes and correlations with tCT_max_

The four fish groups differed significantly in their heart lipid profiles (Table 3). Hexadecanoic acid (C16_0), octadecanoic acid (C18_0), saturated fatty acids (SFA), omega-6 content, α-linolenic acid (C18_3n3) and docosapentaenoic acid (C22_5n3, DPA) did not show significant differences among groups. Monounsaturated fatty acid content was lower in AC than the three other groups. This difference was mainly due to relatively lower palmitoleic (C16_1) and vaccenic acids (C18_1) content in AC. Arachidonic acid (ARA, C20_4n6), eicosapentaenoic acid (EPA, C20_5n3), docosahexaenoic acid (DHA, C22_6n3), omega-3, total PUFA, PI and UI were all significantly higher in AC compared to the three other groups. AC had higher N3/N6 ratios than BC and HA but had similar ratios to HB.

**Fig 3:**
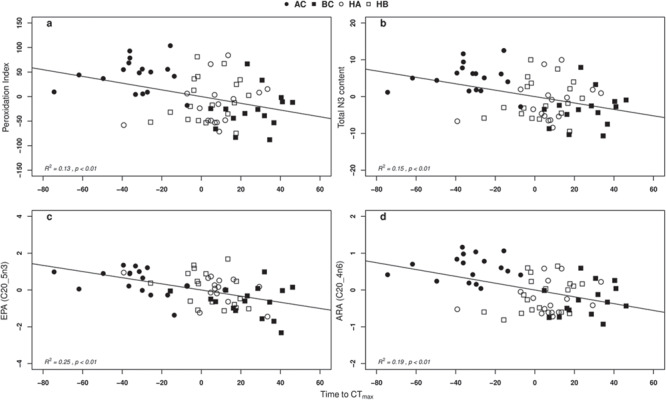
Relationships between PI (a), total omega-3 content (b), EPA (c), ARA (d) and temperature challenge test tCT_max_ in min. All values are expressed as residuals calculated from least linear regressions on body mass. AC (full circles), BC (full squares), HA (empty circles) and HB (empty squares). Equation: (a) PI = −0.69x + 8.74*10^−15^, (b) Total n-3 content = −0.087x + 3.32*10^−16^, (c) EPA = −0.017x + 8,6*10^−17^ and (d) ARA = −0.009x + 3,93*10^−17^.

**Fig 4:**
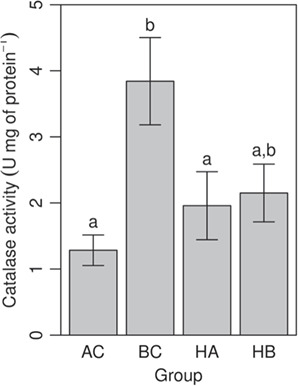
Differences in catalase activity among groups. AC, BC, HA and HB. Values are expressed as means ± SEM. Different letters indicate significant differences (*P < 0.05*).

PI, total n-3 content, EPA and ARA correlated negatively with temperature tolerance (Fig 3a–d,*P <* 0.01). The relationship was most significant for EPA. AC and BC individuals had the most extreme values in the four regression plots, with hybrid groups having values intermediate to the parental groups and slightly closer to BC than to AC. General linear regression analysis with mass as a covariate and AC, BC, HB and HA as group identity confirmed these relationships. The relationships between ARA, PI and total N3 were still significant and we detected no interactions between tCT_max,_ mass or group (Table 2). This also indicates that the correlation with tCT_max_ was dictated by the phenotype of individuals (PI, total N3 content, EPA and ARA) and not the association to one or another taxonomic group. No difference was detected in antioxidant activity among groups except for CAT. CAT activity increased with tCTmax and was highest for BC and HB (Fig. 4, *P < 0,05)*.

**Table 2:** Results for GLM analysis including degrees of freedom (DF), F and *P* value

	PI			N3		EPA		ARA	
	DF	F	*P*-value	F	*P*-value	F	*P*-value	F	*P*-value
tCT_max_	1	6.49	0.014*	7.52	<0.01*	17.55	<0.01*	12.57	<0.01*
mass	1	6.13	0.017*	6.19	0.02*	4.07	0.049*	10.09	<0.01*
group	3	6.03	<0.01*	5.15	<0.01*	0.89	0.45	10.45	<0.01*
interactions									
tCT_max_ *mass	1	0.43	0.51	0.37	0.55	0.34	0.56	1.12	0.29
tCT_max_ *group	3	1.29	0.29	1.08	0.37	0.28	0.84	2.32	0.09
mass*group	3	1.1	0.36	1.19	0.32	1.74	0.17	0.42	0.74
tCT_max_ *mass*group	3	0.48	0.70	0.40	0.76	1.59	0.20	0.78	0.51

Peroxidation (PI), omega-3 content (N3), eicosapentaenoic acid (EPA), arachidonic acid (ARA) and time to critical thermal maximum (tCTmax). *Significant differences (*P* < 0.05).

**Table 3:** Lipid composition of the cardiac muscle of the four groups, AC, BC, HA and HB

	Group																
Fatty Acids	AC				BC			HA				HB				
C16_0	19.35	±	0.62		16.67	±	0.82		16.83	±	0.69		17.85	±	0.72	
C18_0	3.78	±	0.11		3.19	±	0.19		3.30	±	0.15		3.30	±	0.21	
∑ SFA	26.33	±	0.65		25.09	±	1.00		24.95	±	0.73		25.83	±	0.68	
C16_1	4.96	±	0.62	a	8.93	±	1.16	b	7.75	±	0.82	a,b	6.50	±	0.82	a,b
C18_1	13.95	±	0.94	a	19.06	±	1.69	a,b	18.24	±	1.86	a,b	20.76	±	1.47	b
∑ MUFA	20.13	±	0.84	a	29.68	±	1.23	b	26.99	±	1.18	b	26.02	±	1.37	b
C18_2n6	3.20	±	0.18	a	4.36	±	0.29	b	4.61	±	0.24	b	3.93	±	0.27	a,b
C20_4n6 (ARA)	2.46	±	0.09	a	1.56	±	0.11	b	1.63	±	0.11	b	1.76	±	0.11	b
∑ Omega-6	6.16	±	0.21		6.46	±	0.27		6.82	±	0.19		6.07	±	0.25	
C18_3n3	0.72	±	0.11		0.92	±	0.13		0.80	±	0.15		0.60	±	0.14	
C22_5n3 (DPA)	2.49	±	0.12		2.41	±	0.24		2.60	±	0.11		2.40	±	0.21	
C20_5n3 (EPA)	9.30	±	0.18	a	8.25	±	0.22	b	8.87	±	0.17	b	8.87	±	0.21	b
C22_6n3 (DHA)	32.77	±	1.13	a	24.19	±	1.36	b	25.43	±	1.50	b	27.30	±	1.53	b
∑ Omega-3	47.18	±	0.98	a	38.36	±	1.23	b	40.76	±	1.33	b	41.65	±	1.30	b
n-3/n-6	7.80	±	0.32	a	6.15	±	0.42	b	6.10	±	0.33	b	7.11	±	0.41	a,b
∑ PUFA	53.55	±	0.97	a	45.23	±	1.11	b	48.05	±	1.21	b	48.16	±	1.17	b
PI	360.57	±	8.44	a	288.40	±	10.2	b	304.34	±	11.0	b	314.88	±	11.0	b
UI	303.18	±	5.59	a	258.09	±	6.58	b	269.19	±	7.09	b	274.24	±	6.79	b
N	17				15				16				18	

Values are means ± SEM. SFA, saturated fatty acids; MUFA, monounsaturated fatty acids; ARA, arachidonic acid; DPA, docosapentaenoic acid; EPA, eicosapentaenoic; DHA, docosahexaenoic acid; n-3/n-6, omega-3/omega-6 ratio; PUFA, polyunsaturated fatty acids; PI, peroxidation index; UI, unsaturation index. Dissimilar letters indicate significant differences (*P* < 0.05) between groups.

## Discussion

This study provides evidence that membrane robustness towards oxidative stress in the heart muscle is positively correlated with an organism’s capacity to face increasing temperatures.

### Thermal tolerance and body mass

CT_max_ values were within the range of previously reported values for BC and AC (Galbreath *et al.*, 2006; Penney *et al.*, 2014). The lower CT_max_ of AC can partly be explained by their more northerly distribution and adaptation to lower temperature environments (Madeira et al., 2012). Interestingly both hybrid groups performed as well as BC in the temperature challenge test. To our knowledge, these are the first CT_max_ values reported for charr hybrids. Hybridization between closely related species or divergent populations can lead to increased heat tolerance (Edmands and Deimler, 2004; Willett, 2010; Pereira *et al.*, 2014). Genes coming from BC may have an over-dominant effect, causing the superior performance of both hybrid groups over AC. On an evolutionary level, this means that tolerance to temperature increase seems to be a heritable trait and allows the possibility for adaptation. Our results are not surprising as hybrid breakdown resulting from a disruption of mitochondrial and nuclear genome interactions are usually seen in F2 hybrids, not in F1 (Ellison et al., 2008; Barreto and Burton, 2013).

The present results also suggest there is an interaction between body mass and temperature tolerance. Multiple studies have shown decreased performance in heat tolerance for individuals or groups with higher body mass or growth rates (Clark *et al.*, 2008; Daufresne *et al.*, 2009; Roze *et al.*, 2013). A previous study found a weak negative correlation between decreased oxygen levels in arterial blood flow and individual body size. The authors hypothesized that this was caused by poor oxygenation of the spongy myocardium leading to cardiac arrythmia and thus disturbances in blood supply to tissues (Clark et al., 2008). Further research is required to delineate whether the differences of constraints in heart morphology and oxygenation or mitochondrial functions induce variations in performances at high temperatures.

### Fatty acid profiles, antioxidant activity and thermal tolerance

Our study reports a negative correlation between the PI and thermal tolerance. At first thought, it seems that the geographical distribution of the parental species and their specific temperature adaptation could explain this link. However, general linear regression analysis confirmed that when taking into account group identity and body mass as a covariate, this correlation persists meaning that individuals with high PI values in heart tissue are those most affected by increases in temperature. This observation is of particular importance, as we recently showed that a dramatic increase in ROS production at temperatures close to CT_max_ in heart mitochondria of AC could lead to heat-induced heart failure (Christen *et al.*, 2018). Hulbert et al. (2007) advocated a link between PI and oxidative stress tolerance to explain the strong correlation between lifespan and the fatty acid profile divergences among species of endotherms. Munro and Blier (2012) also confirmed this relationship among various marine bivalves. All these studies have revealed a significantly lower PI in long-lived species, supporting the Mitochondrial Oxidative Stress Theory of Aging (MOSTA for a review see (Barja, 2013; Blier *et al.*, 2017). The MOSTA hypothesis suggests that higher content in PUFA may result in higher production of reactive alkyls, following oxidation or peroxidation of membranes (particularly mitochondrial membranes). ROS and reactive alkyls induce denaturation of biomolecules like protein, lipids, and DNA. This accumulation of defective molecules culminates in further dysregulation of mitochondria and potentially in a death spiral (Stern, 2017). It is, however, not clear if traits or mechanisms linking membrane robustness to death from acute heat in fish are the same as those that cause slow physiological deterioration during aging.

Previous studies documented an association between thermal stress and oxidative stress in fish (Kumar *et al.*, 2019). We recently observed in AC that this association prevailed in heart mitochondria (Christen et al. 2018) with a significant upsurge of hydrogen peroxide efflux at a temperature close to CTmax. This finding is in line with the finding of Iftikar et al. (2014) that cardiac mitochondrial dysfunction contributes to heat stress-induced heart failure of *Notolabrus fucicola* and *Notolabrus fucicola*, two wrasses from a cold-temperate and tropical habitat respectively.

Numerous studies have been carried out to pinpoint the mechanisms underlying cardiac failure during acute heat stress. Heart failure seems to be induced by a decrease in heart rate at temperatures close to thermal maxima (Eliason and Anttila, 2017; Ekström *et al.*, 2019). This decline is thought to be caused by ventricular bradycardia which might be induced by changes of the electrical properties and reduced excitability of cardiomyocytes (Haverinen and Vornanen, 2020; Vornanen, 2020). As mitochondria play a key role in cardiac excitability (Rossi *et al.*, 2019) and increases in ROS production is known to decrease excitability (reviewed in: Aggarwal and Makielski, 2013), we hypothesize that mitochondrial dysfunction and the upsurge in ROS efflux of fish heart mitochondria close to thermal limits (Christen *et al.*, 2018) may reduce cardiomyocytes excitability and subsequently induce bradycardia and ultimately result in heart failure. What remains to explore is the connection between PI values or fatty acid profile, ROS production and thermal limits. Do high PI values amplify the impacts of oxidative stress or does it provoke mitochondrial ROS upsurge at lower temperature?

EPA content was also negatively correlated to thermal resistance. Chatelier et al. (2006) and McKenzie et al. (1998) revealed such a negative association between EPA content and physiological traits in seabass (*Dicentrarchus labrax* (McKenzie *et al.*, 1998; Chatelier *et al.*, 2006). The latter studies detected decreased critical swimming speed for individuals displaying higher EPA content in cardiac tissue. This negative relationship of higher EPA content with such traits seems counterintuitive as omega-3, and their metabolites are known to have beneficial effects on human and fish health (Martinez-Rubio *et al.*, 2012; Calder, 2013) and particularly for preventing heart diseases (Innes and Calder, 2020). The beneficial impact of omega-3 results from metabolization of anti-inflammatory metabolites like resolvins by specific enzymes and possibly selenium (Berr *et al.*, 2009), which is independent of the harmful consequences of their oxidative stress-induced degradation. In individuals with high omega-3 content, the negative repercussions of the oxidation of LC-PUFA through oxidative stress might prevail over the beneficial effects of n-3 metabolites during strenuous solicitations of aerobic metabolism. This distress impact of mobilization of maximum aerobic capacity is particularly tangible for species with extremely high omega-3 content like salmonids. As an example, McKenzie et al. (1998) failed to detect a positive relationship between the concentration of cardio-protective metabolites of PUFA, like thromboxane A_2_ and prostacyclin and critical swimming speed performance in seabass (McKenzie *et al.*, 1998). At least for these two metabolites of n-3 degradation, no correlations with critical swimming speed have been detected.

Further work on specific metabolites will help to pinpoint mechanisms involved in heat-induced heart failure. Analytical methods for specific metabolite detection like those of EPA are already available (Kortz *et al.*, 2013; Massey and Nicolaou, 2013). A recent study on ARA’s metabolites (Moon *et al.*, 2017) showed that during heart failure, the activity of enzymes catalyzing the degradation of ARA into proapoptotic metabolites increases. In contrast, the pathway for the cytoprotective metabolites decreases. It would be interesting to test whether this mechanism is involved in heat-induced heart failure in fish.

Total MUFA content was lower in the less heat tolerant AC, which is in line with previous results on fatty acid metabolism and carnitine palmitoyltransferase activity (CPT). Mono-unsaturated fatty acids seem to be preferential fuels for CPT and, subsequently, energy metabolism in various fish species (Sidell *et al.*, 1995). If the lipid profile of the ventricular muscle is similar to those in lipid reserves, the following hypothesis might apply. As energy demand increases with temperature and as fatty acids are essential fuels for ATP production in mitochondria, a lower MUFA content could impose limitations on energy supply at elevated temperatures. This hypothesis is valid only if the availability of MUFA for B-oxidation is reflected in its content, as we measured it in the whole cardiac muscle.

The group with the highest activity of CAT, that is BC, performed better in temperature challenge tests. The higher antioxidant activity might buffer the increased ROS production at temperatures close to thermal limits and reduce the risk of oxidative damage. Of the four antioxidant enzymes assayed, CAT was the only one to correlate with thermal tolerance. Mitochondrial ROS production has long been considered detrimental to organisms leading to cellular dysfunction, senescence, and aging (Trushina and McMurray, 2007; Costantini, 2008; Monaghan *et al.*, 2009; Day, 2014; Mason and Wadley, 2014; Dai, 2017). This perspective is shifting, and some authors now consider mitochondrion as a regulator of ROS content (Munro and Treberg, 2017). Since ROS are important regulators of different pathways, the role of most mitochondrial antioxidant enzymes (SOD, GPx, GSR, and the thioredoxin/peroxiredoxin (Trx/Prx) system) likely evolved to fine-tune the ROS efflux, according to cellular requirements. According to its lower affinity for H_2_O_2_ (Switala and Loewen, 2002), CAT is suspected to buffer H_2_O_2_ during oxidative stress conditions. The correlation of CTmax with only CAT could therefore be explained by its specific function during overflow of H_2_O_2_. This is also in line with the role of ROS management or cell robustness of the heart in the determination of tolerance to temperature increase. Subsequent analysis should, therefore, address whether or not such a positive relationship exists when measuring other antioxidant enzymes activity, such as thioredoxin reductase (TR), glutathione reductase (GR), and total GSH content, in isolated mitochondria (Banh *et al.*, 2015).

## Conclusion

Our study revealed that the PI, omega-3, EPA, and ARA content of the fish heart are all negatively correlated with tolerance to an acute temperature increase. It remains to be validated if the ventricle’s lipid composition is directly linked to acute heart failure during heat stress or if increased susceptibility to peroxidation might affect cardiac physiology on the long-term. Future work should focus on the metabolites of specific fatty acids and on analyzing lipid profiles of mitochondria and mitochondrial subfractions, in order to pinpoint the underlying molecular mechanisms responsible for heat-induced heart failure. Furthermore, quantification of oxidative damage in cardiac tissues of heat tolerant and less tolerant individuals could help to better understand the role of oxidative stress in setting thermal limits. Considering the strong correlation of PI or PUFA content and CT_max_ as well as the putative link between fatty acids susceptibility to peroxidation and ROS induced mitochondrial dysfunctions, additional experiments on the rate of mitochondrial peroxide production are required.

## Funding

This work was supported by Fond de Recherche Québécois Nature et Technologies (P Blier #1664557).
